# Influence of fasting prior to ^18^F-rhPSMA-7.3 (Flotufolastat F-18) PET/CT on biodistribution and tumor uptake

**DOI:** 10.1186/s13550-024-01165-8

**Published:** 2024-11-12

**Authors:** Sonia Grigorascu, Thomas Langbein, Isabel Rauscher, Calogero D’Alessandria, Tobias Maurer, Türkay Hekimsoy, Wolfgang A Weber, Matthias Eiber

**Affiliations:** 1 School of Medicine, Klinikum rechts der Isar, Department of Nuclear Medicine, Technical University of Munich, Ismaninger Str. 22, 81675 Munich, Germany; 2 Department of Urology and Martini-Klinik Prostate Cancer Center, University Hospital Hamburg-Eppendorf, Hamburg, Germany

## Introduction

Prostate cancer (PC) is the most common malignancy in men in the US and Europe [1] and an estimated 30–50% of patients will develop biochemical recurrence (BCR) after the initial curative therapy. Imaging is crucial to guide treatment, both at initial presentation and at BCR. Within the last decade, PSMA (prostate-specific membrane antigen) targeting positron emission tomography (PET) has become the mainstay for localizing recurrent disease given its superior sensitivity compared to conventional imaging modalities [2–4].

PSMA-targeting PET tracers, such as ^68^GaPSMA-11, ^18^F-DCFPyL, and ^18^F-flotufolastat have been approved for initial staging and restaging in the US. Their target, PSMA, is a membrane-bound peptidase which is not only expressed by the prostate and prostate cancer cells but also in other tissues where it is known as Folate hydrolase I or N-acetyl-L-aspartyl-L-glutamate peptidase. In all these tissues PSMA cleaves C-terminal glutamate residues from peptide chains [5–7]. Therefore, the internationally recommended name for PSMA is Glutamate carboxypeptidase II.

Glutamate is an amino acid which is synthesized by the human body and found in various foods. As it has been described as a substrate of PSMA [8], some institutions recommended fasting to prevent potential interference with ligand binding and thus diagnostic performance [8]. An intense PSMA-ligand uptake is typically observed in lacrimal, parotid and submandibular glands and in the small intestine [9, 10]. High oral intake of monosodium glutamate has been described to substantially lower the signal in ^68^Ga-PSMA11 PET of tumor lesions and salivary glands [11].

Dietary restrictions can be straining, especially for patients with concomitant diseases or in poor general condition due to their underlying disease. In addition, long waiting times before the actual scan and dietary restrictions can be problematic for patients, especially the elderly. Currently, in contrast with FDG (fluordeoxyglucose) PET where fasting is essential in clinical routine, patients are not required to fast prior to PSMA-ligand PET. However, there is only limited data available on the impact of fasting vs. non-fasting on PSMA-ligand PET-imaging [12–14].

In this retrospective analysis we investigated whether fasting prior to PET imaging with ^18^F-rhPSMA-7.3 (Flotufolastat F-18) substantially impacts physiologic organ and tumor uptake.

## Materials and methods

### Patients

Data from 60 patients who underwent ^18^F-rhPSMA-7.3 PET/CT (at our institution between September 2018 and September 2021) as part of their routine clinical care were retrospectively reviewed. The analysis was approved by the Ethics Committee of the Technical University Munich (permit 99 –19 S), and is in accordance with the principles of the Declaration of Helsinki. The requirement to obtain informed consent was waived.

We selected patients who underwent PSMA-PET/CT with ^18^F-rhPSMA-7.3 before and after 06/01/2021. Prior to this date, all PET patients were asked to fast for > 6 h prior to arrival at our PET center. For both groups 30 patients (“fasting” vs. “non-fasting” group) with low volume recurrent disease (PSA < 1 ng/ml) were selected because this represents the most challenging clinical situation for PSMA imaging. In addition, the lower tumor burden typically seen in these patients made it unlikely that the results were confounded by a tumor sink effect [15]. Low tumor burden was confirmed by measuring the total radioactivity bound within the tumor lesions delineated by a 50% isocontour of the maximum standardized uptake value (SUV_max_).

### ^18^F-rhPSMA-7.3 synthesis, administration and image acquisition

^18^F-rhPSMA-7.3 was synthesized as recently reported [16]. A median of 288 (IQR: 261–327) MBq ^18^F-rhPSMA‐7.3 were injected as an intravenous bolus with a median of 66 (IQR: 63–72.5) min before PET/CT images were acquired on a Biograph mCT flow scanner (Siemens Medical Solutions, Erlangen, Germany) from vertex-to-mid thigh as recently described [17, 18]. All patients received a diagnostic CT scan after i.v. contrast injection (Iomeron 300, weight-adapted, 1.5 mL/kg) and oral intake of diluted contrast medium (300 mg ioxitalamate [Telebrix; Guerbet]). At tracer administration, 20 mg furosemide were co-injected; patients were asked to void prior to the scan. Images were acquired in 3D mode with an acquisition time of 2.0–2.48 min per bed position (equals 0.8–1.1 table motion). Correction for randoms, dead time, scatter, and attenuation was performed, and images were reconstructed iteratively by an ordered subsets expectation maximization algorithm (four iterations, eight subsets) followed by a smoothing Gaussian filter (5 mm full width at one-half maximum).

### Assessment of biodistribution

SUV_mean_ were determined within standardized 50% isocontour VOIs (volume-of-interest) of the SUV_max_ and a diameter of 30 mm, (salivary glands, lungs, liver, spleen, kidneys, pancreas, duodenum, bladder, bone, muscle, blood pool, and tumor lesions) and compared between both groups. Up to a maximum of 3 lesions per patient were analyzed in decreasing order of the SUV_mean_ and data were averaged. VOI placement and image analyses were performed by one experienced nuclear medicine physician.

### Statistical analysis

The Mann-Whitney U-Test was used to analyze differences between uptake parameters between the “fasting” and “non-fasting” groups. A multivariate analysis (one-way MANOVA) was performed to analyze the effect of fasting on biodistribution. Normal distribution of variables was evaluated by Q-Q plots and the Shapiro-Wilk W test. Data are presented as median (interquartile range), a *P*-value < 0.05 was considered statistically significant. Statistical analysis was performed with SPSS Statistics, version 24 (IBM Corp., USA), and MedCalc, version 14.8.1 (MedCalc Software Ltd., Belgium).

## Results

### Patient population

30 patients were selected for the “non-fasting” and 30 patients for the “fasting” group. Median PSA (0.42 vs. 0.40 ng/ml), median ISUP grade (3 vs. 3), median age and median body weight did not differ between both groups (all *p* > 0.05). The median injected activity/acquisition speed was 298.2 (267.8–330.6) MBq/mm*s^− 1^ for all patients, 289.5 (266.4–317.3) MBq/mm*s^− 1^ for the fasting group, and 316.9 (271.3–345.0) MBq/mm*s^− 1^ for the non-fasting group, respectively. Patient characteristics are presented in Table 1.

**Table 1:** Population data

	Total population (*n* = 60)	fasting group (*n* = 30)	non-fasting group(*n* = 30)	*P* value
Median (IQR) age (y)	70 (63.5–75.5)	72 (65–76)	68.5 (63–75)	0.344
Median (IQR) body weight (kg)	83.5 (74.5–91.5)	79.5 (72–90)	86 (79–92)	0.109
Median (IQR) ISUP grade	3 (2–4)	3 (2–4)	3 (2–4)	0.820
Median (IQR) PSA at timepoint of scan (ng/mL)	0.41 (0.28–0.59)	0.42 (0.30–0.63)	0.40 (0.27–0.48)	0.701
ADT in the 6 months prior to PET/CT	10/60 (16.7%)	3/30 (10%)	7/30 (23.3%)	n/a
Median (IQR) injected activity (MBq)	288 (261–327)	321 (288–349)	261 (237–288)	< 0.001
Median (IQR) uptake time (min)	66 (63–72.5)	68 (64–77)	65 (63–72)	0.216
Median (IQR) aquisition speed (mm/s)	1.1 (0.8–1.1)	1.1 (1.1–1.1)	0.8 (0.8–0.8)	< 0.001
Median (IQR) injected activity /acquisition speed (MBq/mm*s-1)	298.2 (267.8–330.6)	297.25 (266.4–327.3)	312.5(277.5–345.0)	0.277
(estimated) median (IQR) injected ligand mass rhPSMA7.3 [µg]	3.58 (2.5–4.98)	3.78 (2.67–6.37)	3.22 (2.47–4.19)	0.088

### Normal organ biodistribution and tumor lesions evaluated by SUVmean

Median SUV_mean_ in the “non-fasting” group were significantly higher for submandibular glands (23.4 vs. 18.1; *p* < 0.001), pancreas (3.4 vs. 2.6; *p* < 0.001), and duodenum (12.8 vs. 9.8; *p* = 0.028) than in the “fasting” group, respectively. A clear trend towards a higher but neither statistically nor clinically significant median SUV_mean_ in the “non-fasting” group was observed for the parotid gland (18.7 vs. 15.6, *p* = 0.052) and liver (7.8 vs. 6.7, *p* = 0.084), respectively.

Median SUV_mean_ were 2.1 vs. 2.3 (*p* = 0.773) for blood pool, 0.8 vs. 0.7 (*p* = 0.356) for lungs, 7.8 vs. 6.7 (*p* = 0.084) for liver, 9.8 vs. 8.8 (*p* = 0.701) for spleen, 35.4 vs. 33.1 (*p* = 0.209) for kidneys, 1.6 vs. 1.5 (*p* = 0.600) for bone, 0.8 vs. 0.7 (*p* = 0.169) for muscle and 2.4 vs. 2.3 (*p* = 0.554) for bladder in the “non-fasting” vs. “fasting” group, respectively.

**Fig. 1:**
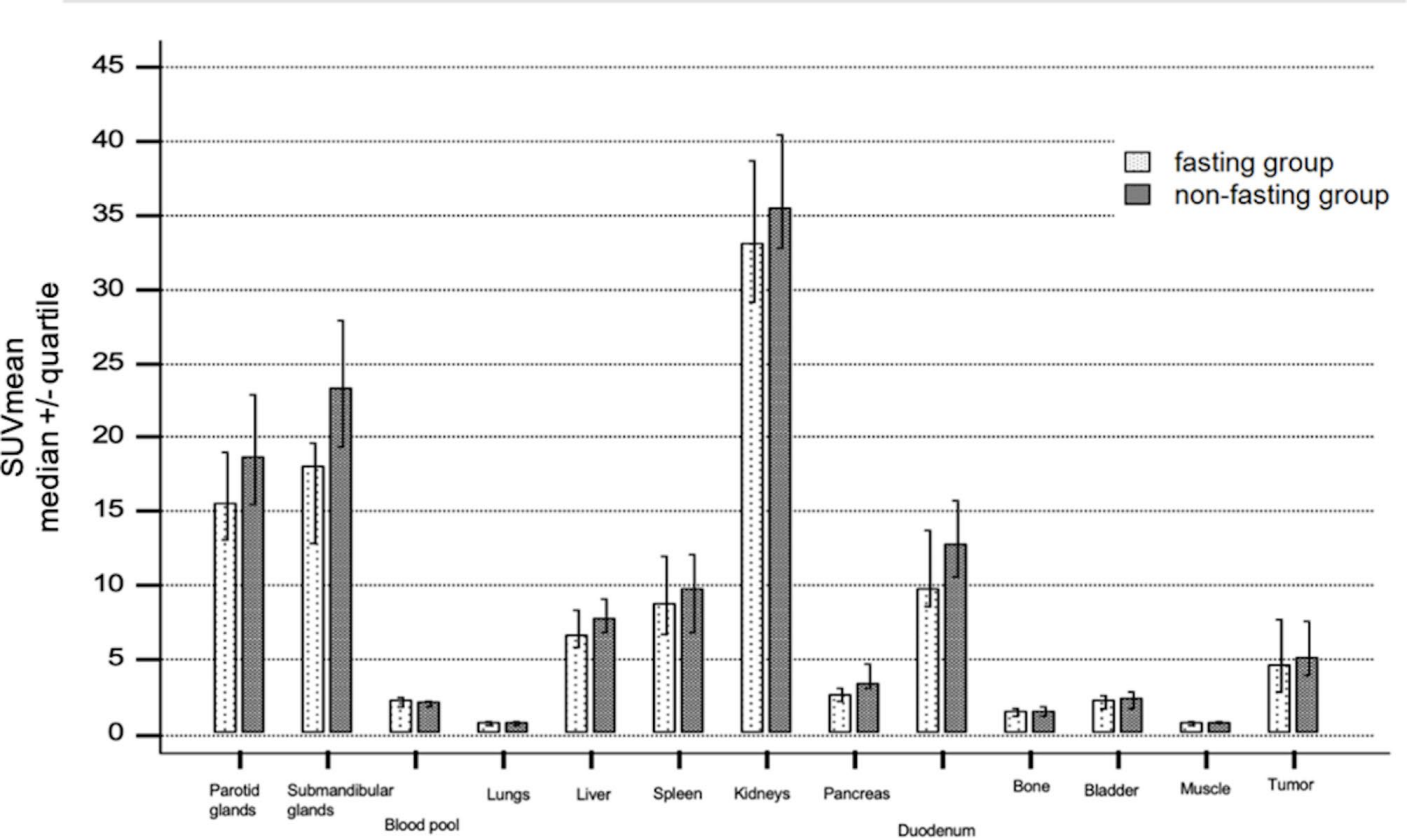
Biodistribution by organ

Median SUV_mean_ for tumor lesions were 5.2 for the “non-fasting” and 4.7 for the “fasting” groups, respectively, and not significantly different (*p* = 0.557). Figure 1 (“Biodistribution by organ”) shows the ^18^F-rhPSMA-7.3 uptake in the “non-fasting” vs. “fasting” group for all organs.

A multivariate analysis (one-way MANOVA) confirmed the statistically significant difference between the “fasting” and “non-fasting” group for the combined dependent variables of tracer distribution, F (5, 54) = 6.123, *P* < 0.001, partial η² = 0.362, Wilk’s Λ = 0.638.

## Discussion

In this retrospective analysis, we investigated whether fasting prior to ^18^F-rhPSMA-7.3 PET-imaging substantially impacts the physiologic organ and tumor uptake for image interpretation. Our data clearly indicate that uptake patterns in most of the organs relevant for clinical reading and in malignant lesions are not substantially different between fasting and non-fasting patients. Small, and for some organs, statistically significant differences were observed in organs that are related to digestive functions such as salivary glands, pancreas, duodenum, and liver (Table 2). In those organs, ^18^F-rhPSMA-7.3 uptake was consistently higher in patients who did not fast prior to tracer injection. We hypothesize that digestive stimulation of these organs leads to greater blood flow and therefore higher tracer accumulation as the main explanation for this phenomenon.

**Table 2:** Biodistribution by organ

Tissue	fasting group Median SUV_mean_ (IQR)	non-fasting group Median SUV_mean_ (IQR)	*P* value
Parotid glands	15.6 (13.2–19.0)	18.7 (15.6–23.0)	**0.052**
Submandibular glands	18.1 (12.9–19.7)	23.4 (19.4–27.9)	**< 0.001**
Bloodpool	2.3 (1.8–2.5)	2.1 (1.9–2.3)	0.773
Lungs	0.7 (0.6–0.8)	0.8 (0.7–0.9)	0.356
Liver	6.7 (5.9–8.4)	7.8 (6.9–9.2)	**0.084**
Spleen	8.8 (6.7–12.0)	9.8 (6.8–12.2)	0.701
Kidneys	33.1 (29.2–38.7)	35.4 (32.8–40.5)	0.209
Pancreas	2.6 (2.2–3.2)	3.4 (3.1–4.7)	**< 0.001**
Duodenum	9.8 (8.7–13.7)	12.8 (10.6–15.8)	**0.028**
Bone	1.5 (1.3–1.7)	1.6 (1.3–1.9)	0.600
Bladder	2.3 (1.8–2.6)	2.4 (1.8–2.8)	0.554
Muscle	0.7 (0.6–0.8)	0.8 (0.7–0.9)	0.169
Tumor	4.7 (2.9–7.8)	5.2 (4.0–7.7)	0.557

Postprandial hyperemia of the gastrointestinal system is a phenomenon caused by multiple factors, like tissue oxygen tension and nutrient absorption [19]. Food intake has been proven to cause changes in the intestinal tissue [20], e.g., increased splanchnic blood flow and oxygen uptake [21, 22], which are suspected to occur due to an increased secretory and absorptive activity of the gut and muscular tissue during intestinal contractions [23]. Food properties regarding composition, e.g., fat, carbohydrate, and protein percentage, are also said to have different impacts on the hyperemia increase [24]. The correlation between blood flow, specifically of the tumor, and ^68^Ga-PSMA-11 uptake has been studied in a 2021 paper by Jochumsen et al., where the authors hypothesized that PSMA-ligand uptake in tumors is limited by, and also a reflection of, tumor blood flow [25]. The combination of increased gastrointestinal blood flow after food intake and blood flow-related PSMA-ligand accumulation is likely to explain the higher activity levels in our non-fasted patients.

Effects of dietary preparations on PSMA-ligand PET have also been investigated in the past. Rahbar et al. [12] analyzed uptake characteristics of ^18^F-PSMA1007 in 40 patients with different dietary preparations (fasting > 6 h, fasting > 6 h plus highly caloric drink 1 h before injection, fasting > 6 h plus highly caloric drink 1 h after injection, no restrictions at all) [12]. The authors’ main aim was to reduce physiological organ uptake, especially in the liver and small bowel to improve the potential tumor-to-background signal. The rationale was translated from the use of highly caloric diets on liver uptake in cardiac scintigraphy with ^99m^Tc-methoxy isobutyl isonitrile [26]. However, in this investigation, no impact of dietary preparations and fasting on tracer uptake in the liver and small bowel was observed.

The effects of fasting on ^18^F-DCFPyL uptake were investigated in a study conducted by Wondergem et al. [13]. PET/CT scans of a fasting (> 6 h) and a non-fasting cohort of 50 and 48 PC patients, respectively, were analyzed for differences regarding tracer accumulation. Similar to our results higher uptake levels were present for the submandibular gland, liver, duodenum, and other gastro-intestinal organs with either a significant statistical difference or a clear trend. Finally, Mohan et al. investigated the effect of gustatory stimulation on ^18^F-DCFPyL uptake in the salivary glands of 10 PC patients. All patients had previously undergone a clinically indicated ^18^F-DCFPyL PET/CT scan [14]. A second scan was performed within one month with gustatory stimulation achieved by intake of saliva-inducing food items rich in sugar, acid, and fats shortly before and up to 10 min after tracer injection. The rationale for this analysis was based on the use of salivary gland stimulation in radioiodine treatment in thyroid cancer to shorten radioactivity transit time. Results showed a clear increase, though non-significant, in tracer uptake in the salivary glands.

The salivary glands are known to be vulnerable to radiation and often show high tracer accumulation after PSMA-targeted radionuclide therapy (PRLT). This can lead to mild xerostomia in about 30% of mCRPC patients undergoing Lu-177-radionuclide therapy [27, 28] and is the main dose-limiting factor in Ac-225-radionuclide therapy. In a retrospective analysis by Feuerecker et al. [29], irreversible grade 1/2 xerostomia was observed in 26 (100%) patients after the first cycle of Ac-225-PSMA and led to early treatment termination for six (23%) patients. The exact mechanism of this side effect is still unknown and few effective strategies to prevent it have been found so far. Because salivary gland toxicity is currently the main dose-limiting factor for PRLT [30], reducing or even eliminating sialotoxicity is of utmost interest. Our findings could have clinical meaning regarding the prevention of xerostomia using therapeutic PSMA-targeted radioligands.

There are several limitations in our study. We retrospectively selected patients imaged before and after an internal transition date when we stopped asking patients to fast prior to arrival. Additional changes were implemented regarding injected activity as lower amounts were injected after this date (3.0 MBq per kg body weight vs. 4.0). However, SUV calculation is independent of injected activity and uptake time and the use of SUV_mean_ (as opposed to the SUV_max_) is unlikely to be substantially influenced by injected activity. The qualitative image interpretation with respect to the influence of the fasting state was not performed with a systematic matrix, but by visual assessment. However, results from our quantitative data only show substantial differences in the biodistribution for salivary glands and pancreas, supporting the lack of differences. Finally, it is important to mention that we analyzed a small patient group of 30 patients per cohort. It is therefore that the lack of an observed difference in tumor uptake is related to the small sample size. Furthermore, by selecting patients retrospectively, there is no certainty as to whether everyone adhered to the fasting instructions. It would be interesting to reapply these instructions to a prospective study design with a possibly larger cohort to definitely confirm and reaffirm our findings.

## Conclusion

Dietary restrictions before ^18^F-rhPSMA-7.3 PET have no clinically relevant impact on biodistribution and especially on the uptake of tumor lesions. Based on these data fasting does not seem to be necessary prior to ^18^F-rhPSMA-7.3 PET.

## Data Availability

The datasets generated during and/or analyzed during the current study are available from the corresponding author SG upon reasonable request.

## Abbreviations

Ac-225: Actinium-225
BCR: Biochemical recurrence
CT: Computer tomography
FDG: Fluordeoxyglucose
ISUP: International Society of Urological Pathology grading system
Lu-177: Lutetium-177
mCRPC: Metastatic castration-resistant prostate cancer
PC: Prostate cancer
PET: Positron emission tomography scan
PRLT: Prostate radionuclide therapy
PSMA: Prostate-specific membrane antigen
PSA: Prostate-specific antigen
rh: Radiohybrid
SUV: Standardized uptake value
VOI: Volume-of-Interest
